# Supplementary material for: Single-molecule imaging of chromatin remodelers reveals role of ATPase in promoting fast kinetics of target search and dissociation from chromatin
Source: eLife. 2021 Jul 27;10:e69387. doi: 10.7554/eLife.69387 (PMC8352589; doi:10.7554/eLife.69387)
Supplement: Figure 5—figure supplement 1—source data 1. [file elife-69387-fig5-figsupp1-data1.zip › Figure5-sourcedata2.pdf]

Related to Figure 5-supplement 1A
Uncropped gel images

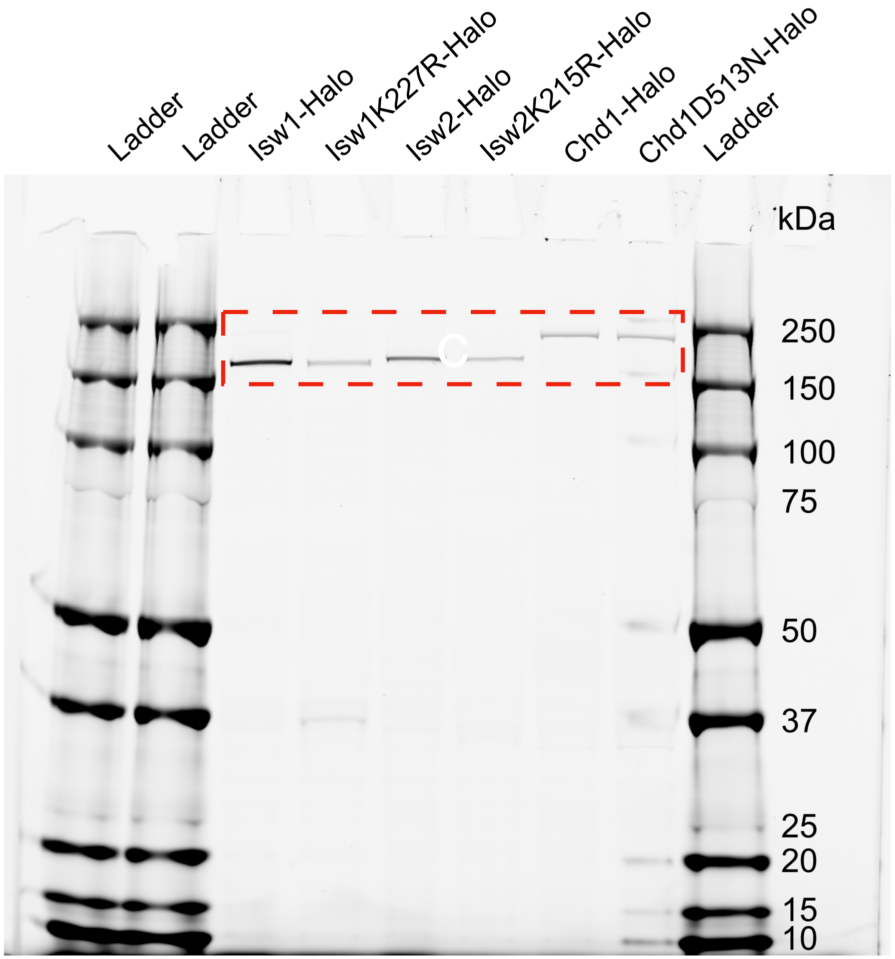

Typhoon5
Cy5 channel excitation [550 PMT]

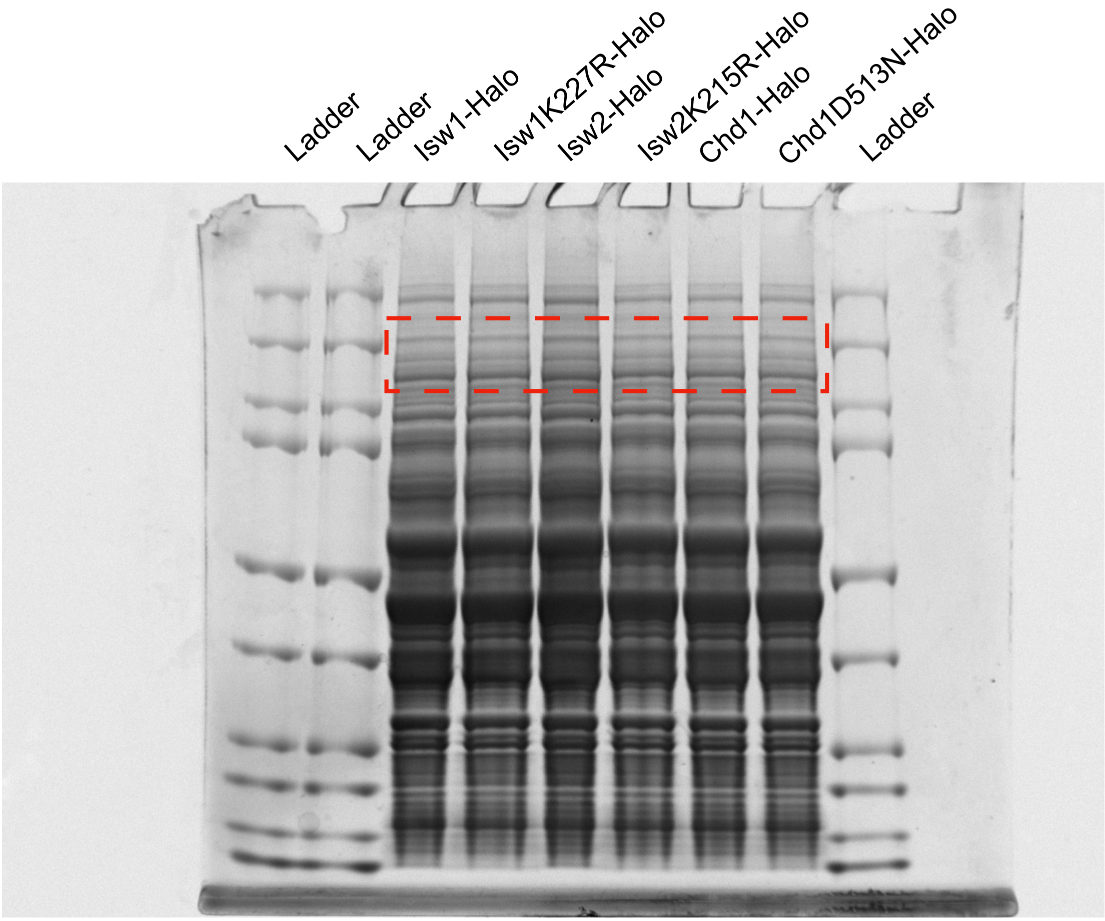

Coomassie dye staining
(loading control)
